# Combinatorial therapy of chitosan hydrogel-based zinc oxide nanocomposite attenuates the virulence of *Streptococcus mutans*

**DOI:** 10.1186/s12866-021-02128-y

**Published:** 2021-02-24

**Authors:** Shima Afrasiabi, Abbas Bahador, Alireza Partoazar

**Affiliations:** 1 Department of Microbiology, School of Medicine, Tehran University of Medical Sciences, Tehran, Iran; 2 Oral Microbiology Laboratory, Department of Microbiology, School of Medicine, Tehran University of Medical Sciences, Tehran, Iran; 3 Experimental Medicine Research Center, Tehran University of Medical Sciences, Tehran, Iran

**Keywords:** Dental caries, *Streptococcus mutans*, Biofilms, Chitosan, Zinc oxide, Nanocomposites

## Abstract

**Background:**

Biofilm formation is an important causative factor in the expansion of the carious lesions in the enamel. Hence, new approaches to efficient antibacterial agents are highly demanded. This study was conducted to evaluate the antimicrobial-biofilm activity of chitosan hydrogel (CS gel), zinc oxide/ zeolite nanocomposite (ZnONC) either separately or combined together [ZnONC / CS gel (ZnONC-CS)] against *Streptococcus mutans* biofilm.

**Results:**

MTT assay demonstrated that the ZnONC-CS exhibits a non-cytotoxic effect (> 90% cell viability) toward human gingival fibroblast cells at different dosages (78.1–625 μg/mL) within 72 h. In comparison with CS gel and ZnONC, ZnONC-CS was superior at biofilm formation and metabolic activity reduction by 33 and 45%, respectively; (*P* < 0.05). The field emission scanning electron microscopy micrographs of the biofilms grown on the enamel slabs were largely in concordance with the quantitative biofilm assay results. Consistent with the reducing effect of ZnONC-CS on biofilm formation, the expression levels of *gtfB*, *gtfC*, and *ftf* significantly decreased.

**Conclusions:**

Taken together, excellent compatibility coupled with an enhanced antimicrobial effect against *S. mutans* biofilm has equipped ZnONC-CS as a promising candidate for dental biofilm control.

## Introduction

Dental caries is one of the multi-factorial diseases affecting people worldwide. Deep caries lesions can increase the risk of dental pulp infection. Carious lesions initiate where oral biofilms are allowed to grow and remain on the tooth surface [1]. In general, the biofilm is a complicated matrix containing proteins and exopolysaccharides (EPS), providing a stable shelter for bacterial cells that is hard to remove [2]. Treatment of dental biofilms is a serious challenge due to their strong adhesion to the surface of teeth and tendency to calcify into dental calculus [3].

*Streptococcus mutans* is one of the primary microbial culprits of dental caries [4]. The cariogenic potential associated with *S. mutans* includes adhesion to tooth surfaces, synthesis of EPS via glucosyltransferases (Gtfs), stimulates biofilm formation, and the ability to use sucrose and generate an acidic environment [5]. The bacterium produces three Gtfs, −B, −C and –D, whose cooperative action is necessary for bacterial adhesion to the tooth surface [6]. GtfB and -C, which utilize the glucose moiety of sucrose as the substrate to produce extracellular glucose polymers, are encoded by the *gtfB* and *-C* genes, respectively [7]. *S. mutans* also produces a fructosyltransferase (Ftf), which synthesizes extracellular fructans from the fructose moiety of sucrose [8]. They can act as binding sites for bacterial aggregations [9].

The bacterial composition of dental biofilms is mainly formed in areas that are protected from the forces on dental biofilms reduction by the tongue, cheeks, and tooth brushing [10]. Mechanical approaches and antimicrobial agents for controlling dental biofilms are limited by adverse effects such as damage of oral mucosa, dysgeusia, burning sensation, ulcerative lesions, and enamel erosion and therefore, are not suitable for long-term usage [1, 2]. Fluoride is a well-known antiplaque agent via the demineralization/remineralization process. However, an adverse effect like fluorosis may occur if the fluoride excessive use [11]. Therefore, early intervention with minimal side effects should be considered to control the dental biofilm formation.

Despite the novel function of nanoparticles (NPs) that have been acted rather than their bulk materials, the aggregation of NPs usually is an unavoidable problem [12, 13]. Nanocomposites (NCs) have been employed as versatile tolls to control the major limitations in growing NPs size and stabilization [14]. Zinc oxide nanomaterials are recognized to be Generally Regarded As Safe (GRAS) by the Food and Drug Administration (FDA) [15] which have several advantages such as safety as well as anti-biofilm and antimicrobial activities in a broad spectrum of microorganisms. Small size and increased surface/volume ratio of NPs permit them to penetrate or interact with bacterial membranes, resulting in cell membrane destruction, leakage of intracellular contents, and finally bacterial death [16, 17].

Zeolite (Ze) is a microporous crystalline material of aluminosilicate, which can slowly release pre-loaded antimicrobials over long periods [17, 18]. Zinc oxide NPs (ZnO-NPs) doped Ze (ZnONC) showed a wide range of antibacterial properties against Gram-positive and Gram-negative bacteria [14, 17]. Moreover, chitosan (CS) is a natural linear polycationic biopolymer with good biocompatibility, biodegradability, low toxicity, and antibacterial activity that has adhesion properties to mucosal surfaces [19]. Bioadhesive characteristics of CS would prolong its contact time with the treatment site and further availability of the release content [20].

The CS hydrogel (CS gel) form causes its long-term storage and more application in different formulations [21]. According to the ability of free amino groups in the CS compound that perfectly complexes with metals and metal oxide NPs, there is a novel strategy to use the combination polymers with other nano-sized metal oxides such as ZnO NPs as the antimicrobial agent [22, 23]. However, there is no any information about the beneficial effects of CS gel in combination with ZnONC (ZnONC-CS) on *S. mutans* to obtain a synergistic effect for better antibacterial activity so far. In that sense, this study aimed to combine the CS gel with ZnONC to attenuate *S. mutans* virulence properties as well as their effect on the biocompatibility in vitro.

## Results

### MICs of CS gel, ZnONC, and ZnONC-CS

ZnONC, ZnONC-CS (both at 78.1–1250 μg/mL) and CS gel (156.2–2500 μg/mL) significantly inhibited *S. mutans* growth when compared to control (*P* < 0.05). Lower concentrations of ZnONC and ZnONC-CS (both at 2.4–39 μg/mL) and CS gel (4.8–78.1 μg/mL), also affected *S. mutans* growth, but this was not statistically significant (*P* > 0.05). Therefore, the MIC of ZnONC, ZnONC-CS and CS gel was 78.1, 78.1 and 156.2 μg/mL, respectively.

### In vitro cytotoxicity assay of CS gel, ZnONC, and ZnONC-CS

**Fig. 1:**
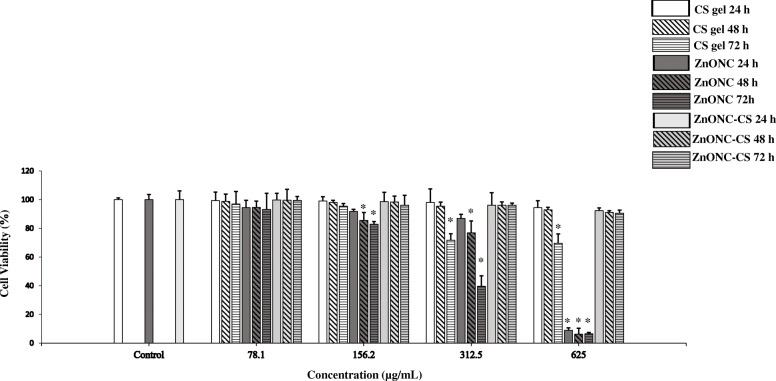
Cell viability of human gingival fibroblast cells (HGFs) after treatment with chitosan hydrogel (CS gel), zinc oxide nanocomposite (ZnONC), and chitosan/zinc oxide nanocomposite hydrogel (ZnONC-CS) with different concentrations up to 72 h. The values were recorded as total number of living cells. Error bars represent standard deviation. * *P* < 0.05 compared to the untreated sample

The cytotoxic effects of CS gel, ZnONC, and ZnONC-CS were evaluated with HGFs in vitro. The cell viability was measured for extraction media of materials mentioned above with different concentrations (concentration is shown in Fig. 1) up to 72 h. According to the results, the CS gel toxicity is increased upon increasing the concentration of CS gel (more than 156.2 μg/mL) and incubation up to 48 h didn’t show any significant effect on cell viability. As it can be seen in Fig. 1, the cell viability did not change significantly in the low concentrations of ZnONC up to 78.1 μg/mL (at all times; *P* > 0.05) while the cell viability was decreased (*P* < 0.05) with increasing concentration (156.2–625 μg/mL) and/ or exposure time (48 and 72 h) of the nanocomposite. As seen in Fig. 1, the ZnONC-CS did not change the cell viability upon changing the incubation time which shows the biocompatibility of ZnONC-CS. The ZnONC-CS treated cells decreased the cytotoxicity compared to ZnONC under similar conditions. The viability was determined greater than 90% up to the concentration of 625 μg/mL of ZnONC-CS at all time points (*P* > 0.05). The permissible limit of cytocompatibility is considered to be > 75%, according to ISO standards 10,993–5:2009. Therefore, ZnONC-CS can be considered a safe product in this stage.

### Effects of CS gel, ZnONC, and ZnONC on *S. mutans* biofilm formation ability inhibition

The results of this study demonstrated that 39 μg/mL (½ MIC) of ZnONC-CS decreased 33% of biofilm formation of *S. mutans* (*P* = 0.00). In contrast, Biofilm formation of *S. mutans* was not significantly reduced with 78.1 and 39 μg/mL (both at ½ MIC) of CS gel and ZnONC (3.15 and 14.23%, *P* = 0.91 and 0.07, espectively). Besides, the statistical analyses indicated a significant difference between the ZnONC-CS group and other treatment groups (CS gel and ZnONC), [*P =* 0.00 and 0.02, respectively], however, no differences were found between the CS gel group and ZnONC group (*P* = 0.18). These results provide evidence that the ZnONC-CS can significantly affect the biofilm formation of *S. mutans* (Table 1).

**Table 1:** Comparative data of subinhibitory concentrations related to chitosan hydrogel (CS gel), zinc oxide nanocomposite (ZnONC), and chitosan/zinc oxide nanocomposite hydrogel (ZnONC-CS) effects on biofilm formation and metabolic activity of *Streptococcus mutans*

Experiments		Biofilm formation		Metabolic activity	
		% reduction	***P*** value	% reduction	***P*** value
CS gel	vs. Control	3.15	0.91	19.75^a^	0.00
ZnONC		14.23	0.07	30.00^a^	0.00
ZnONC-CS		33.00^a^	0.00	45.00^a^	0.00
ZnONC	vs. CS gel	11.08	0.18	10.25	0.06
ZnONC-CS		29.85^a^	0.00	25.25^a^	0.00
ZnONC-CS	vs. ZnONC	18.77^a^	0.02	15.00^a^	0.00

^a^Significant statistical differences between groups

### Assessment of metabolic activity

As shown in Table 1, the metabolic activity of *S. mutans* after treatment with CS gel, ZnONC, and ZnONC-CS at sub-MIC concentration was decreased up to 19.75, 30, and 45%, respectively. These results show that ZnONC-CS can significantly affect the metabolic activity of *S. mutans* (*P* = 0.00). The data suggest that the ZnONC and CS gel at sub-MIC concentration does not significantly inhibit the biofilm formation, but metabolic activity is affected.

### Fe-SEM of biofilm formation ability inhibition

**Fig. 2:**
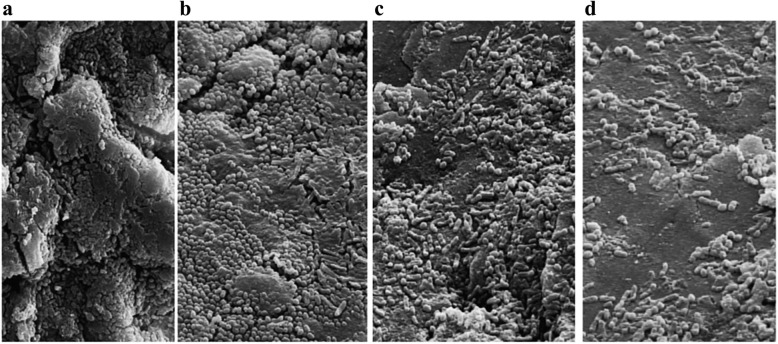
Scanning electron micrograph (3000× magnification) of *Streptococcus mutans* biofilm. **a** Untreated biofilm control, **b** Biofilms were grown on human enamel slabs surface in the presence of sub inhibitory concentration (sub-MIC) of chitosan hydrogel (CS gel), **c** zinc oxide nanocomposite (ZnONC), and **d** chitosan/zinc oxide nanocomposite hydrogel (ZnONC-CS)

In the present work, a study was performed to visualize the effect of CS gel, ZnONC and ZnONC-CS on the architecture of the *S. mutans* in the biofilm. For this purpose, the cell structure of treated and untreated cells was investigated using Fe-SEM, and the results presented are in Fig. 2. The untreated bacterial cells of *S. mutans* demonstrated that cells with large clusters embedded in an EPS (Fig. 2a). When biofilm was treated with a sub-MIC concentration of CS gel and ZnONC, the numbers of bacteria were reduced but not very impressive (Fig. 2b, c). A more substantial decrease in the number of cells and single cells or short chains was observed when the biofilm was treated with ZnONC-CS (Fig. 2d).

### *gtfB, −C,* and *ftf* gene expression reduction by ZnONC-CS

**Fig. 3:**
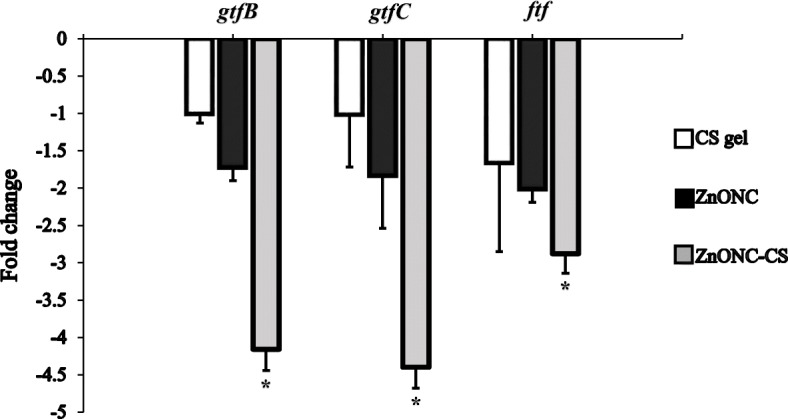
Fold change in mRNA expression level of *gtfB, gtfC* and *ftf* gene from *Streptococcus mutans* after treatment with chitosan hydrogel (CS gel), zinc oxide nanocomposite (ZnONC), and chitosan/zinc oxide nanocomposite hydrogel (ZnONC-CS). Error bars represent standard deviation. * *P* < 0.05 compared to the control

The antibacterial activities of CS gel, ZnONC, and ZnONC-CS against *S. mutans* were further confirmed by quantitative Real-Time PCR (Fig. 3). After treatment of *S. mutans* with different experimental groups, the profile of gene expression was determined. As the results reveal, the *gtfB, −C,* and *ftf* gene expression profiling downregulated in *S. mutans* cells 1.0, 1.02, 1.81- and 1.73, 1.84, 1.49- fold following CS gel and ZnONC, respectively. The expression level of *gtfB, −C,* and *ftf* reduced 4.16, 4.40, and 2.88-fold in ZnONC-CS. Based on the results, the level of gene expression in ZnONC-CS compared to control was remarkably different (*P* < 0.05), while no significant differences were found in the expression of mentioned genes following CS gel and ZnONC groups (*P* > 0.05).

## Discussion

The bacterial biofilms associated with infected carious dentine are a global public health problem [24]. The bacteria embedded in biofilms display a set of ‘emergent properties’ that differ noticeably from the planktonic lifestyle [25]. Dental caries is a consequence of oral microbial dysbiosis. Although several therapeutic strategies including, antimicrobial peptides, probiotics, bacteriophages have produced encouraging effects to reverse dysbiosis, the development of new and effective strategies is an urgent need to control biofilm expansion [24, 26].

Metal oxide NPs such as ZnO-NPs are reported to induce reactive oxygen species (ROS) and the cells exposed to oxidative stress which damage cellular components [27]. In a previous study, it has been revealed that the ZnO-NPs modification with polymeric materials reduces their cytotoxicity. Furthermore, CS can mask the NPs and subsequently, preventing the release of Zn^2+^ ions and ROS [28]. Our findings showed that ZnONC-CS has a stronger antibacterial effect along with lower cytotoxicity compared to CS gel or ZnONC. It suggests that CS plays an important role in the enhancement of antibacterial activity against *S. mutans* and reduction of cell cytotoxicity on HGFs cells. In accordance with the evidences extracted of this study, Mehta et al. have reported that combination of CS with ZnO nanomicelles (CZNPs) shows to be more effective against multiple drug resistance (MDR) Gram-positive biofilms than CS or ZnO alone. Moreover, CZNPs have been established as relatively non-toxic compared to both ZnO and CS NPs alone [29].

In the current study, CS gel and ZnONC at their sub-MIC concentration had slightly anti-biofilm activity on *S. mutans* (with an inhibition rate of 3.15 and 14.23%, respectively). In our previous study, anti-biofilm trait of ZnONC against *Enterococcus faecalis* was investigated, and findings indicated that ZnONC at concentrations of 1.25–2.5 mg*/*mL never had any significant effect on biofilm formation of *E. faecalis* [17]. ZnONC-CS has been shown to have higher anti-biofilm potency than the CS gel and ZnONC at sub-MIC concentrations. As an explanation, the higher antibacterial activity of ZnONC-CS than the CS gel and ZnONC can be attributed to the facilitated diffusion of particles into cells and decreased agglomeration [30]. Actually, the combination of CS with NPs has improved the antibacterial activity by increasing the positive charge density of CS amine group leading to better complexation efficiency with anionic molecules of cell surface [30]. Furthermore, the Fe-SEM of saliva-coated enamel slabs confirmed that the cluster of bacterial cells was more shattered by ZnONC-CS. Although the ability for the biofilm reduction in *S. mutans* did not at the same rate as metabolic activity, suggesting that viable cells remaining inside the biofilm decreased their metabolic activity.

In the present study, ZnONC-CS cause more decrease in the expression of *gtfB, −C,* and *ftf* gene than that of CS gel or ZnONC alone. This result was in accordance with Badawy et al., who reported that prepared CS/ZnONC causes a significant decrease in biofilm formation of *Pseudomonas aeruginosa*. Also, CS/ZnONC causes more decrease in the expression of *Las*I and *Rhl*I genes of *P. aeruginosa* than exposure to the CS alone [31]. Based on the results presented here, it is obvious that the antibacterial activity of ZnONC-CS is greater than that of CS or ZnONC alone. Therefore, ZnONC-CS could be potential therapeutic agents for attenuating the *gtfB, −C,* and *ftf* activity known virulence attributes of *S. mutans*. Moreover, the current results of this study showed that *gtfC* was affected in the presence of ZnONC-CS more than the other examined genes. Highly homologous *gtfB*, and -*C* gene, resulting in an impressive decrease in the generation of water-insoluble glucans [32].

Overall, the results of the present study showed that ZnONC-CS has a reinforcing effect on cariogenic virulence factors of *S. mutans* along with lower cytotoxicity compared to other groups. Also, the antimicrobial effects of ZnONC-CS should be assessed on multispecies dental biofilms and further studies are needed for the full understanding of the performance and safety of this formulation in vivo studies. Therefore, concerning limitation of this study, new investigations need to verify the clinical relevance of these results. Finally, Our findings suggest that ZnONC-CS could potentially use as an anti-biofilm agent in mouth rinse formulations and oral healthcare products.

## Conclusion

Our findings revealed that the inhibitory effect of ZnONC-CS accelerates reduction of the biofilm formation and cariogenic properties of *S. mutans* rather than ZnONC or CS alone. The biocompatibility of ZnONC-CS in vitro assessment was improved using its effective concentration that suggests the clinical prospects of this nanohydrogel in the control of dental biofilms.

## Methods

### Preparation of ZnONC

ZnONC was synthesized and characterized in our previous study [17]. Briefly, zeolite powder was shacked in a round bottom flask containing deionized water (D.W) for 1 h, and filtered before being dried at 80 °C. The Zn^2+^ exchanged zeolites were performed by impregnation of 10 g of zeolite powder into 7 g of a Zn (acetate) 2. 2 H2O aqueous solution under stirring conditions at 60 °C for 1 h. For fabrication of ZnO nanoparticle on the zeolite bed, a solution of NaOH 1 M was added to the suspension to obtain pH = 12. After 2 h, the composite materials were collected by filtration and was washed thoroughly with D. W to remove the excess zinc, and dried in the oven at 80 °C. Then the product was calcined for 2 h at 400 °C. The ZnONC was analyzed by x-ray diffraction, x-ray fluorescence and field emission scanning electron microscopy (Fe-SEM) coupled with energy dispersive x-ray. The results revealed that the morphology of the ZnONC is spherical with an average size of 30 nm [17].

### Preparation of CS solution

A CS (Sigma, USA) stock solution (1 g/ 100 mL) was prepared in 1% (v/v) acetic acid and the mixture solution was subjected to constant stirring with a magnetic stirrer at ambient temperature overnight. Then, the CS solution was sterilized in an autoclave (121 °C, 15 min) [33].

### Preparation of ZnONC-CS

Five mL of CS solution was mixed with 5 mg of ZnONC for 60 min at 37 °C using a magnetic stirrer. The resultant light-yellow viscous solution turns into white precipitate with slow addition of NaOH (1 M) until the pH reached 8.0–8.5. The final product was kept at 4 °C to settle down for 24 h. Similarly, a blank CS hydrogel was prepared as mentioned above without the ZnONC content.

### Bacterial strain and culture conditions

*S. mutans* ATCC 35668 was obtained from the Iranian Biological Resource Center (Tehran, Iran) and grown in brain–heart infusion broth (BHI; Laboratorios Conda, Torrejón de Ardoz, Spain) aerobically (5% CO_2_) at 37 °C for 24 h.

### Cytotoxicity assessment

Human gingival fibroblasts (HGFs; IBRC C10459) purchased from the Iranian Biological Resource Center (Tehran, Iran) were cultured in Dulbecco’s modified Eagle’s medium (DMEM; Biowest, France) supplemented with 10% fetal bovine serum (Gibco, UK) and pen-streptomycin (Biowest, France). The cells were seeded into the 96-well microtiterplate at a density of 10,000 cells/well followed by overnight incubation. Parallel to this experiment, a range of concentrations from 78.1 to 625 μg/mL of CS, ZnONC and ZnONC-CS were shaken in an incubator for 24, 48, and 72 h in DMEM to prepare extraction media. The seeded medium was replaced with 200 μL of the extraction media followed by incubation for 24 h. Post incubation, the cells were washed with fresh sterile phosphate-buffered saline (PBS) to eliminate non-adherent cells and media. Finally, a 3-(4,5-dimethylthiazol-2-yl)-2,5-diphenyltetrazolium bromide (MTT) assay kit (Sigma-Aldrich) was used to determine the cytotoxicity in HGF cells at 570 nm according to the manufacturer’s instructions [34]. Dimethyl sulfoxide (DMSO) at 10% concentration served as the cell death control (positive control). The permissible limit of cytotoxicity effect is considered to be > 75% according to ISO standards 10,993–5:2009 [35].

### Determination of the minimum inhibitory concentrations (MICs)

The MIC of the ZnONC, CS gel, and ZnONC-CS against *S. mutans* was determined by the microdilution method as recommended by the Clinical and Laboratory Standards Institute guidelines [36]. Briefly, 100 μL of BHI broth was added to the well of a round-bottom 96-well microtiterplate, and 100 μL of ZnONC, CS gel, and ZnONC-CS (all stock solution = 5 mg/mL) was then added to the first well in column 1, 2, and 3, respectively. They were diluted to 1:2. Afterward, 100 μL of *S. mutans* suspension (1.5 × 10^6^ CFU/mL) was added to each dilution and incubated at 37 °C for 24 h in 5% CO2. Following incubation, the contents of each well were serially diluted and plated onto BHI agar plates (Merck, Darmstadt, Germany) and incubated for 48 h at 37 °C in 5% CO2. Subsequently, the CFU/mL was calculated using the method of Breed et al. [37]. MIC was interpreted as the lowest level concentration of the products at which bacterial growth was inhibited. Sub-MIC values were one dilution lower than MIC values and were applied for evaluation of their ability to abolish *S. mutans* virulence activity.

### Biofilm formation evaluation by crystal violet

Quantification of the biofilm formation ability of *S. mutans* was performed according to a previous study [38]. Briefly, 200 μL aliquots of *S. mutans* cells suspended in planktonic cultures at a final concentration of 1.5 × 10^5^ CFU/mL were transferred to flat-bottomed 96-well microtiterplate. Bacterial cells were treated with ZnONC, CS gel, and ZnONC-CS at sub-MIC level, and the plate was incubated for 48 h at 37 °C in 5% CO2 to allow for biofilm formation. After incubation, the microplate contents were emptied out from each well and washed three times with PBS to remove the unadhered bacteria. The cells in the biofilm were stained for 15 min with 200 μL of crystal violet (0.1%, w/v). After washing thrice with PBS, the bound dye was eluted with 150 μL of 95% ethanol under mild shaking, and absorbance at 550 nm was determined using a microplate reader (Thermo Fisher Scientific, US).

### XTT-reduction assay

The metabolic activity of treated cells with CS gel, ZnONC, and ZnONC-CS was determined by the reduction of sodium 3-[1-(phenylamino-carbonyl)-3, 4-tetrazolium]-bis (4-methoxy-6-nitro) benzene sulfonic acid hydrate (Roche Applied Science, Indianapolis, IN, US), as previously described [39]. One hundred microliters of culture (1.5 × 10^5^ cells/mL) was dispensed in 96-well microtiterplate supplemented with sub-MICs of CS gel, ZnONC, and ZnONC-CS until 24 h at 37 °C. Afterward, the prepared solution of the XTT solution (50 μL) was added to each well and mixed thoroughly. The plate was incubated in the dark at 37 °C for 3 h. The reduced formazan-colored was spectrophotometrically measured using a microplate reader at 492 nm.

### Fe-SEM imaging

To mimic the biofilm environment, an ex vivo study was performed to investigate the effect of ZnONC, CS gel, and ZnONC-CS on the structure of 48 h grown biofilms on human enamel slab (3 mm × 3 mm × 1 mm). Saliva was collected from a healthy volunteer and then centrifuged at 8000 g for 15 min at 4 °C. Each enamel slab was placed in 200 μL of the sterilized saliva of a 24-well microtiterplate. The plate was then incubated at 37 °C for 2 h to coat the human enamel slabs with a salivary pellicle. Post incubation, the human enamel slabs were carefully washed with PBS. Saliva-coated enamel slabs were suspended into the 96-well microtiter plate containing 200 μL of *S. mutans* suspension (1.5 × 10^5^ CFU/mL) treated with CS gel, ZnONC, and ZnONC-CS at sub-MIC concentration. Saliva-coated enamel slabs treated with BHI broth used as the control. The biofilm was grown on these saliva-coated human enamel slabs for 48 h. At the end of this incubation period, the medium was discarded, and the biofilms were fixed using methanol and then dehydrated in increasing concentrations of ethanol (20, 40, 60, 80, and 100%). The human enamel slabs were finally dried, then mounted, and sputter-coated with a thin layer of gold-palladium and then investigated by Fe-SEM (HITACHI S-4160, Japan).

### *gtfB*, *gtfC*, and *ftf* gene expression under the planktonic condition

In the current study, the changes of *gtfB*, *gtfC*, and *ftf* gene expression of *S. mutans* were analyzed in different treatment groups according to the study design. Briefly, the *S. mutans* ATCC 35668 strain grown in BHI broth in presence of CS gel, ZnONC, or ZnONC-CS. An untreated sample was used as the control. Subsequently, the total RNA was extracted at the middle of the exponential phase of growth (Treatment duration ~ 6 h; PH: 6.5) using the RNX-plus solution (SinaClon, Iran) according to the manufacturer’s instructions. Traces of genomic DNA were removed using the RNase-free DNase I treatment (Thermo Scientific GmbH, Deutschland, Germany). The amount and quality of extracted RNA were based on the 260/280-nm ratio measured using a NanoDrop spectrophotometer (Thermo Fisher Scientific, US). cDNA synthesis was performed by RevertAid First Strand cDNA Synthesis Kit (Fermentas), according to the manufacturer’s instructions. Quantitative real-time PCR (qRT-PCR) was performed with a Line-GeneK Real-Time PCR Detection System (Bioer Technol-ogy, Hangzhou, China). A sequence of primers used in this research: *gtfB* F: 5′-TGTTGTTACTGCTAATGAAGAA-3′; *gtfB* R: 5′-GCTACTGATTGTCGTTACTG-3′, *gtfC* F: 5′- GAGTTGGTATCGTCCTAAGT-3′; *gtfC* R: 5′-CTGGTTGCTGTATTGTATGTT-3′, *ftf* F: 5′- ACGGCGACTTACTCTTAT-3′; *ftf* R: 5′-TTACCTGCGACTTCATTAC-3′, *16S rRNA* F: 5′-GCAGAAGGGGAGAGTGGAAT-3′; *16S rRNA* R: 5′- GGCCTAACACCTAGCACTCA-3′ [40]. The mRNA levels were quantified in relation to endogenous control gene coding for *16S rRNA*. Changes in expression levels of target genes were analyzed using the Eq. 2^-ΔΔCt^ [41].

### Statistical analysis

All these experiments were done at least three times and the values are expressed as mean ± standard deviation. The commercial software SPSS version 26 was used for statistical analyses.

Statistical analysis was performed using the independent-samples *t*-test to compare two groups. Differences among more than two groups were analyzed by one-way ANOVA followed by the Tukey HSD post hoc test, with the significance level set at 0.05.

## Data Availability

All documents and additional data are available from the corresponding author upon reasonable request.
